# Conventional versus endoscopic components separation technique: New anthropometric calculation for selection of surgical approach

**DOI:** 10.3906/sag-1708-112

**Published:** 2019-08-08

**Authors:** Yaşar Subutay PEKER, Oğuz HANÇERLİOĞULLARI, Mehmet Fatih CAN, Sezai DEMİRBAŞ

**Affiliations:** 1 Department of General Surgery, Gülhane Training and Research Hospital, University of Medical Sciences, Ankara Turkey; 2 Department of General Surgery, TOBB ETÜ University Hospital, Ankara Turkey

**Keywords:** Components separation index, components separation technique, giant ventral incisional hernia, Gülhane index for components separation

## Abstract

**Background/aim:**

Giant ventral incisional hernias (GVIHs) are hard to manage for surgeons. This problem was resolved in 1990 with the components separation technique (CST). We aimed to compare endoscopic and conventional CST for GVIHs and find a new anthropometric calculation.

**Materials and methods:**

In this prospective nonrandomized clinical trial, 21 patients were treated with endoscopic or conventional CST between 2012 and 2016. Eight patients (38.1%) were operated endoscopically and 13 (61.9%) conventionally on the basis of preoperative tomography results, hernia surface area (HSA), number of recent abdominal operations, comorbidities, and the presence or history of ostomy. Groups in which prosthetic material was applied were also compared with groups in which it was not.

**Results:**

There was no statistically significant difference between endoscopic and conventional CST groups in terms of complications. A weakly statistically significant difference (P = 0.069) was found between the components separation index (CSI) of mesh-applied and not-applied patients. HSA/body surface area (BSA) was statistically significantly different between endoscopic and conventional CST groups.

**Conclusion:**

According to our results, HSA/BSA and CSI are statistically successful for preoperative prediction of mesh placement. Furthermore, HSA/BSA preoperatively successfully predicts whether conventional or endoscopic CST should be used in patients with GVIH.

## 1. Introduction

The incidence of incisional hernias varies between 0.5% and 20% in the adult population [1–5]. This frequency has increased, especially with major abdominal surgeries, but with increasing medical knowledge and technology the incidence of incisional hernia is gradually diminishing with the development of new abdominal closure techniques and new suture materials.

**Figure 1:**
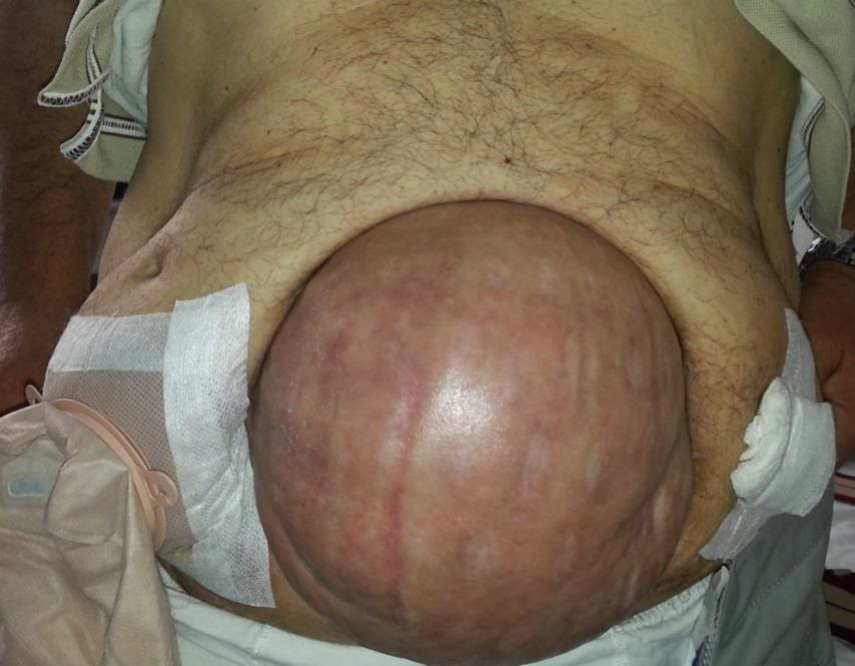
A case of GVIH in which the anatomy of the anterior wall of the abdomen is completely impaired.

Although incisional hernias are considered manageable for surgeons, the real problems for surgeons begin with giant ventral incisional hernias (GVIHs), as shown in Figure 1. In complicated cases in which the integrity of the anterior wall of the abdomen is nonexistent, primary repair cannot be considered because of its high rates of complications and recurrence and because it cannot provide sufficient support for the anterior abdominal wall [6–8]. Prosthetic materials used in such cases may occasionally lead to more complicated and hard-to-manage cases, such as enterocutaneous fistulas. In addition, the patient’s postoperative quality of life is impaired by the inability to reconstruct the erectile function of the abdominal wall. In these cases, aside from the commonly used methods, the preferred method is the components separation technique (CST) [9,10].

CST used in GVIH is a method of primary suturing. Contrary to classical data, CST in GVIH is recommended as a primary suturing method [11]; recommendations against primary suturing in GVIH have been revised in light of updated information. The difference between CST and classical primary suturation is that CST provides no-tension reconstruction of the linea alba with massive medial mobilization of abdominal rectus muscles by transecting the fascia of external oblique abdominal muscle at the semilunar line without transecting the fascia of internal oblique and transverse abdominal muscle. Thus, compared to classical primary repairs, the use of prosthetic material is greatly reduced. The method, which was first described in 1990 by Ramirez et al. [12], was developed and modified over time to address its 20% rate of postoperative wound infections and 18.2% rate of recurrence within 1 year [1,11]. These modifications include preservation of rectus muscle perforators, minimal subcutaneous dissection, application of prosthetic material support to the primary repair line if necessary, and finally, endoscopic CST [11].

When CST was defined by Ramirez et al. [12] in the early 1990s, they could not have predicted that this method would develop so quickly over a period of about 25 years. In 2012, Rulli et al. first described gasless and single-port techniques, in which endoscopic technique and modified technique for giant inguinoscrotal hernia are described [13]. In addition to these modifications, in 2013 CST was described as the “Components Separation Endoscopic and Subcutaneous Approach” by Daes et al. in South America [14].

In this prospective nonrandomized study, we aimed to compare the postoperative results of the most commonly applied modifications of CST: conventional and endoscopic CST for the treatment of GVIHs. While comparing the two groups, we also aimed to find a new anthropometric calculation for applying or not applying mesh, and to preoperatively predict the type of surgery.

## 2. Materials and methods

In the General Surgery Clinic of Gülhane Training and Research Hospital, patients with GVIH who applied for surgery between March 2013 and March 2016 were treated with CST. Twenty-one patients were included in this study. Patients who were older than 18 years of age, had a history of at least one abdominal laparotomy, and had GVIH, were included in the study. Patients’ performance scores, comorbidities, and sex were not considered as exclusion criteria. Patients who were younger than 18 years and who could be treated with primary suturation were excluded from the study.

Intravenous/oral contrast-enhanced computed tomography (CT) was performed to plan the surgeries. Before surgery, the body mass index (BMI) of the patients was calculated by measuring their height and weight; body surface area (BSA) was calculated with the Mosteller formula; and hernia surface area (HSA) was calculated using CT images by mathematical field formulas. After data collection, patients were scheduled for endoscopic or conventional CST considering HSA, number of recent abdominal operations, comorbidities, and presence or history of ostomy. Descriptive statistics on continuous data are presented as mean, standard deviation, median, minimum, and maximum values; for discontinuous data, percentage values are given. The Mann–Whitney U test was used to evaluate the difference between the two surgical types for quantitative values, and Fisher’s exact test was used to compare discontinuous data. The differences in visual analogue scale (VAS) values of the endoscopic and conventional CST groups on the first, third, and seventh day were evaluated by Fisher’s exact test [15–17]. SPSS 11.5 (SPSS Inc., Chicago, IL, USA) was used for statistical analysis and calculation. Microsoft Excel 2010 (Microsoft Co., Redmond, WA, USA) was used for collection of patient data. This study was approved by Ankara University Ethics Committee on 11 March 2013, serial number 04–185–13.

## 3. Results

Endoscopic CST was applied in 8 patients (38.10%), and conventional CST was applied in 13 patients (61.90%). Twenty-one patients, 16 (76.19%) male and 5 (23.80%) female, were included in the study. The mean age of the patients was 43.57 ± 19.99 years, and the median age was 50 years (20–82). The mean age of the patients who underwent endoscopic CST was 32.75 ± 17.14 years, and the median age was 22.5 years (20–61). The mean age of the patients who underwent conventional CST was 50.23 ± 19.21 years, and the median age was 56 years (21–82). A statistically significant difference was found between the mean age of the two groups (P = 0.037).

The mean BMI of the patients was calculated as 26.39 ± 5.59 kg/m², and the median was 25.71 kg/m² (17.99–37.46); according to the World Health Organization (WHO), this BMI classification was in the preobese category. The maximum BMI was 37.46 kg/m², thus class 2 obese patients also underwent CST for GVIH treatment. The mean BMI for endoscopically operated patients was 25.62 ± 5.90 kg/m², and the median was 23.36 kg/m² (20.06–37.11); the mean BMI for conventionally operated patients was 26.85 ± 5.58 kg/m², and the median was 27.55 kg/m² (17.10–37.46). No statistically significant differences were found between the two groups (P = 0.414).

BSA was calculated using the Mosteller formula. The mean BSA was 1.86 ± 0.15 m², and the median was 1.88 m² (1.57–2.11). The mean BSA of endoscopically operated patients was 1.90 ± 0.10 m², and median was 23.36 m² (1.80–2.50). Conventionally operated patients had a mean BSA of 1.84 ± 0.15 m², and the median was 1.88 m² (1.57–2.11). Statistical analysis revealed no significant difference between the two groups (P = 0.645).

Patients were evaluated for the size of the GVIH defects (in centimeters). In 21 patients, hernias with a mean maximum width of 12.10 ± 3.95 cm and a median of 12 cm (5–21) were closed. Hernia defects closed with endoscopic CST were a mean of 10.63 ± 2.76 cm and median of 10.25 cm (6–15), with a mean unilateral rectus relaxation of 5.57 cm and median of 5.13 cm (3–7.5). The mean width of linea alba defects closed with conventional CST was found to be 13.00 ± 4.38 cm with a median of 13 cm (5–21); the mean unilateral rectus relaxation was 6.5 cm with a median of 6 cm (2.5–10.5). There was no statistically significant difference between the techniques in terms of ability to close hernia defects (P = 0.185).

**Figure 2:**
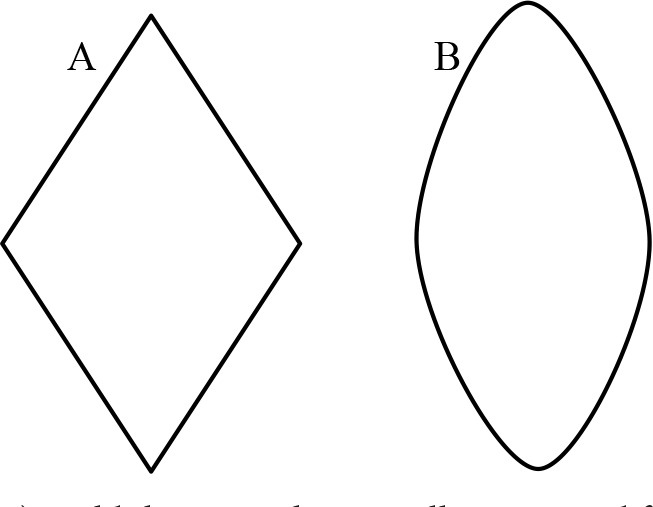
A) Mold that is mathematically accounted for area. B) Compatible mold.

HSA was calculated according to the mathematical formula. However, because the hernia defect is convex at the sides, 10% is added to the calculated surface area, as shown in Figure 2. The mean HSA was 0.16 ± 0.08 m², with a median of 0.16 m² (0.02–0.40). The mean HSA of the patients who were treated with endoscopic CST was 0.13 ± 0.03 m², and the median was 0.13 m² (0.07–0.19); the mean HSA of the patients who were treated with conventional CST was 0.18 ± 0.09 m², and the median was 0.18 m² (0.02–0.40). A weakly statistically significant difference was found between the two groups (P = 0.064).

The percentage of HSA/BSA was calculated to evaluate what proportion of BSA was herniated; the mean was 8.73% ± 4.09% and the median was 8.17% (1.13–19.07). Patients who underwent endoscopic and conventional CST were compared in terms of HSA/BSA percentage. The mean was 6.65% ± 1.78%, and the median was 6.48% (3.66–9.99) in patients who were treated with endoscopic CST. The mean was 10.02 ± 4.62% and the median was 10.34% (1.11–19.07) in patients who were treated with conventional CST. A statistically significant difference was found between the groups using the Mann–Whitney U test (P = 0.045). This anthropometric measurement, described here for the first time, is named the Gülhane Index for Components Separation (GICS).

**Figure 3:**
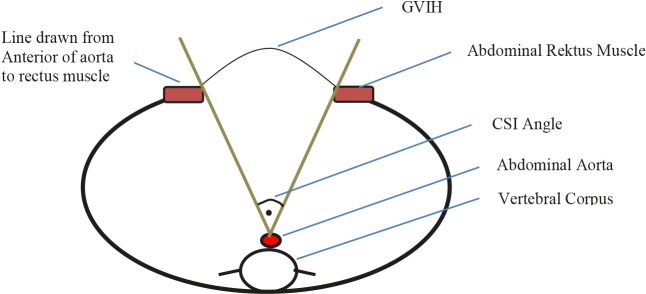
Schematic CT section for CSI angle calculation.

Patients were evaluated for their components separation index (CSI) (18). On the preoperative abdominal CT, the slice of CT on which the hernia width is largest and the angle at the intersection of the straight lines drawn from the medial edges of both rectus muscles to the anterior of the abdominal aorta was calculated. Subsequently, the CSI was calculated by dividing the angle by 360 as shown in Figure 3. The mean CSI angle was 68.23° ± 28.70°, with a median of 60° (27–130). The mean CSI after dividing the angle by 360 was 0.19 ± 0.08, with a median of 0.17 (0.08–0.36). Patients were compared in terms of CSI, and no statistically significant difference was found between the two groups using the Mann-Whitney U test (P = 0.104) as shown in Table 1.

**Table 1:** CSI comparison of patients with ECST and CCST.

Surgery type	N	Mean	SD	Median	Min	Max	P-value
Conventional	13	0.21	0.088	0.19	0.077	0.36	0.104
Endoscopic	8	0.15	0.049	0.15	0.075	0.29	
Total	21	0.19	0.79	0.17	0.075	0.36	

When the VAS values on the first, third, and seventh day were compared for both groups using the Mann–Whitney U test, there was a weakly statistically significant difference on day 1 and no significant differences on days 3 and 7 (P = 0.064, P = 0.135, and P = 0.270, respectively), as shown in Table 2.

**Table 2:** Comparisons of 1st, 3rd, and 7th day VAS of patients operated with ECST– CST.

	1st day VAS		3rd day VAS		7th day VAS
	Mean ± SD	Median(min–max)	Mean ± SD	Median(min–max)	Mean ± SD
Total	5.14 ± 2.19	5 (2–9)	2.75 ± 1.65	3 (1–7)	1.40 ± 1.35
Conventional	5.77 ± 2.35	6 (2–9)	3.25 ± 1.86	3 (1–7)	1.75 ± 1.54
Endoscopic	4.13 ± 1.55	5 (2–6)	2.00 ± 0.93	2 (1–3)	0.88 ± 0.84
P-value	0.064		0.135		0.270

Patients included in the study were evaluated for hospitalization time. The mean hospitalization time was assessed for 20 patients because the patient who died on the second postoperative day was not evaluated. Patients had a mean hospitalization duration of 8.30 ± 2.87 days with a median of 7 days (5–13). The mean hospital stay for patients who underwent endoscopic CST was 8.25 ± 3.06 days with a median of 7 days (5–13); mean hospital stay for those who underwent conventional CST was 8.33 ± 2.87 days with a median of 7.5 days (5–13). There was no statistically significant difference between the two groups (P = 0.910).

Patients were evaluated for postoperative complications, which were divided into two categories: related to the surgical field and nonsurgical field. A total of 7 patients (33.33%) had postoperative complications: 4 (19.05%) had complications related to the surgical field, whereas 3 (14.29%) had nonsurgical field complications, as shown in Tables 3 and 4. There was no statistically significant difference between the two groups according to postoperative complications (P = 0.656), as shown in Table 5.

**Table 3:** Surgical site-related complications and treatments.

Surgical field related complications and treatments
Ischemia on wound lips - revision made
Cellulite - strained by antibiotherapy
Hematoma - drained
Seroma - drained

**Table 4:** Nonsurgical field complications and treatments.

Out-of-surgical complications and treatments
Enterocutaneous fistula - patient underwent staged reoperations
MI - patient died
Ileus - patient underwent bridectomy

**Table 5:** The results of postoperative complication development according to surgery type.

		Total complications		Total
		–	+	
Surgery type	Conventional	8 (61.5%)	5 (38.5%)	13 (100.0%)
	Endoscopic	6 (75.0%)	2 (25.0%)	8 (100.0%)
Total		14 (66.7%)	7 (33.3%)	21 (100.0%)

The mean follow-up period was 928.90 ± 310.85 days and the median was 965.50 days (39–1228) for a total of 20 patients. The patient who died on the second postoperative day was not evaluated in terms of the follow-up period.

The Mann–Whitney U test was used to determine whether there was a statistically significant difference in the BMI of the patients in whom prosthetic material was applied and those in whom it was not applied. There was a weakly statistically significant difference between the two groups (P = 0.058), as shown in Table 6. Patients also were evaluated for CSI according to prosthetic material use. The statistical evaluation using the Mann–Whitney U test revealed a weakly statistically significant difference between patients in whom prosthetic material was applied and those in whom it was not (P = 0.069), as shown in Table 7.

**Table 6:** BMI (kg/m²) of patients according to the use of prosthetic material.

Prosthetic material	N	Mean	SD	Median	Min	Max	P-value
+	9	23.86	4.15	23.05	19.38	32.30	0.058
–	12	28.28	5.93	27.98	17.99	37.46	
Total	21	26.39	5.59	25.71	17.99	37.46	

**Table 7:** CSI of patients according to the use of prosthetic material.

Prosthetic material	N	Mean	SD	Median	Min	Max	P-value
–	9	0.15	0.06	0.15	0.08	0.28	0.069
+	12	0.22	0.09	0.20	0.08	0.36	
Total	21	0.19	0.08	0.17	0.08	0.36	

Hernia recurrence (8 × 7 cm) was detected in one patient (4.76%) who underwent conventional CST and received prosthetic material. Exitus occurred in one patient (4.76%); cardiopulmonary arrest (myocardial infarction) and/or cerebrovascular event were considered to be the cause of the exitus on postoperative day 2. This 82-year-old patient had a history of hypertension, diabetes mellitus, colon adenocarcinoma, and underwent conventional CST.

## 4. Discussion

In GVIH, conventional CST has been used for 28 years [12], whereas endoscopic CST was much more recently developed in 2000 [19]. When a literature survey was conducted with the keywords “components separation” and “components separation hernia” using the search engines PubMed and Web of Science with EndNote X7 8.4 (Thomson-Reuters, 2015), fewer than 1000 results were found. Some of the results are case presentations and some are technical discussions; studies related to this new technique are still very scarce.

As in our study, 8 studies in the literature survey compared conventional and endoscopic CST in treating uncommon GVIHs [15,20–26]. One is a study that mainly emphasizes both methods in terms of cost [25]. Jensen et al., who prepared the first review of the subject in 2014 [27], searched PubMed and Embase for the component separation technique and found 222 studies. When duplications were excluded, they found only 5 studies that compared endoscopic and conventional methods. All the studies they reviewed were retrospective. In 2014, however, Azoury et al. reported a study of 76 patients, 42 endoscopic and 34 conventional CST [21]. In the same year, Azoury et al. published another study of 42 patients who underwent endoscopic CST and reported that they treated hernia defects with the open method in 17 patients and with the laparoscopic method in 25; they collected data retrospectively [28]. According to the aforementioned literature survey, our study is one of the largest prospectively comparing conventional CST with endoscopic CST in GVIHs. In addition, the mean follow-up period of our patients was 928.90 ± 310.85 days, and the median was 965.50 days (39–1228), which is also one of the longest follow-up periods for this subject reported in the literature. 

Although a statistically significant difference was not found when comparing patients with endoscopic CST and conventional CST in terms of BMI, we found that the mean BMI of both groups was preobese according to the WHO classification. We performed endoscopic CST in a class 2 obese patient with the highest BMI in our series (37.11 kg/m²). Of the 6 patients who were obese with a BMI over 30 kg/m², 2 underwent endoscopic CST and 4 underwent conventional CST. Complications did not develop in patients with endoscopic CST. The major complication of enterocutaneous fistula developed in one obese patient who was treated with conventional CST. During the literature survey, we found a statistically significant difference between endoscopic and conventional CST in terms of BMI only in Azoury et al. [21]. In other studies, we found that the BMI of those who were selected for the conventional method was higher than in those selected for the endoscopic method, although there was no statistically significant difference, as in our study. In the literature review, the highest BMI among patients who underwent endoscopic CST for GVIH was 46 kg/m², as reported by Todd et al. [29]. As a result, we think that obesity is not a relative contraindication in terms of endoscopic or conventional CST. Furthermore, only Fox et al. reported that the risk of recurrence in patients with BMI > 35 kg/m² was statistically significant (P = 0.05) [22].

Christy et al. reported a retrospective series of 36 patients who underwent CST in 2012 [18]. Patients were divided into two groups: prosthetic material applied (n = 18) or not applied (n = 18). In the study, CSI was calculated to predict whether the prosthetic material should or should not be applied to the defective hernia area preoperatively after CST. Similarly, Lahiri et al. used anthropometric measurements to predict complications preoperatively in 2016 [30]. Christy et al. found a statistically significant difference in CSI between the groups; the authors reported that CSI could predict the use of prosthetic material in CST preoperatively (P < 0.001), but the study reported no threshold value for prosthetic material application. We tried to apply the anthropometric measure of Christy et al. for preoperative endoscopic or conventional CST selection prediction instead of for prosthetic material selection. As a result, no statistically significant difference in CSI was found between the two groups (P = 0.104), but the P-value is just above the threshold level for weak significance. For this reason, we think that CSI is an anthropometric index that surgeons can use for selecting endoscopic or conventional CST, although larger studies are needed. In addition, we confirmed the validity of CSI of Christy et al. in our study and compared the CSI of those patients in whom prosthetic material was not applied with those in whom it was applied (application of prosthetic material was not because of postoperative presence of hernia defect but rather because the surgeon considered hernia sutures to be too tight or noted a lack of adequate tissue support). As a result, we found a statistically weak difference between the two groups in terms of CSI (P = 0.069). Finally, we think that the anthropometric index found by Christy et al. is valuable for predicting the use of prosthetic material in patients with GVIH after CST, although it is not as strong in our study as that in their study.

To contribute to the study by Christy et al., we compared patients with and without prosthetic material applied in terms of BMI and found a statistically weak difference between the two groups (P = 0.058). In line with these data, we think that in patients who will undergo CST, BMI can be used as an alternative to CSI to predict the application of prosthetic material.

As Lahiri et al. and Christy et al. did anthropometric calculations, we calculated the HSA/BSA ratio of our patients as an anthropometric measurement, taking into account the patients’ biometric differences and evaluating each patient with his or her own biometric characteristics to standardize the preoperative choice of conventional or endoscopic method in patients with GVIH. When the HSA/BSA ratio of both endoscopic CST and conventional CST groups were compared, a statistically significant difference was detected (P = 0.045). The maximum HSA/BSA ratio was 9.99% for endoscopic CST patients and 19.07% for conventional CST patients. As a result, we think that the HSA/BSA ratio is an anthropometric measurement that can be used for preoperatively selecting endoscopic or conventional CST in GVIHs. In the literature survey, we saw that these data have not been studied in any previous publications. This anthropometric measurement, which we call the GICS, should be studied with wider studies and may also be applied for other types of hernia, such as lumbar hernia.

All patients had complete closure of the hernia defects after the CST operation in our study. For this reason, we statistically analyzed how much rectus medialization of CST was achieved using preoperative measurements of hernia width and determined whether there was any difference between endoscopic and conventional methods. We found that defects with a mean size of 13.00 cm (unilateral 6.50) and 10.63 cm (unilateral 5.57) were closed using conventional and endoscopic methods, respectively. When both groups were statistically analyzed for their ability to mobilize the rectus muscle, no significant difference was detected (P = 0.185). When we investigated the literature for how much rectus muscle mobilization is provided by CST, we found reports of up to 10 cm unilaterally with a total of 20 cm of defect closure provided by conventional CST and no data given for endoscopic CST [1,11]. In our study, we obtained a maximum unilateral rectus muscle mobilization of 10.5 cm with 21 cm defect closure by conventional CST, thus confirming the data in the literature. With the endoscopic method, we found that a maximum of 15 cm of the defect were covered. The literature does not yet mention how much defect can be closed with endoscopic CST. To our knowledge, we are the first to report that endoscopic CST can close GVIH defects of up to 15 cm and provide 7.5 cm unilateral rectus mobilization.

The most important reason for the development of endoscopic CST is to avoid the postoperative wound complications of conventional CST that occur in giant skin grafts created during surgery and to reduce early postoperative pain. Patients who were treated with endoscopic and conventional methods in our study were compared in terms of postoperative complications and pain. Results showed that postoperative pain with endoscopic CST was similar to that with conventional CST on the third and seventh days and was partially superior to conventional CST in the early postoperative pain on the first day. Both groups were also compared in terms of postoperative complications; there was no statistically significant difference between them (P = 0.656). Many studies have reported that the greatest advantage of the endoscopic method compared with the conventional method is a reduction in postoperative complications, especially related to the wound site [15,20,22–26], but Azoury et al. found no significant difference in postoperative complications between the methods. We also did not find this advantage of the endoscopic procedure to be statistically significant in our study.

Regarding complications, endoscopic CST has been reported to be more advantageous than conventional CST, and although we cannot prove this difference statistically, major complications were reported after conventional CST. After conventional CST, a semilunar hernia that developed as a natural result of the technique, weakening the semilunar line was reported for only one patient [31]. In this case, reported by Mackay et al. in 2008, the semilunar (spigelian) hernia that developed on postoperative day 6 was repaired with polypropylene suture material and reinforced with mesh. It is necessary to discuss whether the integrity of the aponeuroses of the internal oblique and transverse abdominal muscles is preserved during the dissection of the aponeurosis of the external oblique muscle in CST among patients with a history of poor general condition and multiple surgeries. Mackay and colleagues did not address this issue in their study. In our series, semilunar herniation did not develop after components separation.

As a conclusion to our discussion, we want to draw attention to a particular topic. Although we discuss data that are relevant to patients, it is important to note that a substantial portion of the data is actually obtained through anthropometric measurements of the patient; a significant portion of these measurements have also been made using CT measurements. In addition, CT of GVIHs also shows the herniated organs and the status of the abdomen, allowing the surgeon to avoid encountering intraoperative surprises. For this reason, we suggest that GVIH patients be evaluated with CT preoperatively if there is no contraindication. In addition, as indicated by Blairve et al. in 2015, preoperative CT can determine the need for CST and assess postoperative complications by measuring abdominal wall thickness and hernia size [32].

Patients with GVIH are difficult for surgeons to manage, but with CST, this group of patients is becoming more manageable. However, the lack of experience related to CST, and therefore the lack of studies, has caused surgeons to be timid in applying this new method. Furthermore, the fact that patients with GVIHs are few in number prevents surgeons from having sufficient experience. This is also the weak point of our work, but as we increase our experience, we will continue to expand our study and transfer our knowledge.

In conclusion, we believe that endoscopic CST is a feasible technique and may be applied instead of conventional CST. However, we suggest that GICS and CSI be calculated preoperatively to aid decision-making in whether to apply endoscopic or conventional CST. GICS has a more statistically significant P-value than CSI for predicting the type of surgery. Finally, we support the conclusion reached by Christy et al., who stated that CSI is important for preoperatively deciding whether to apply mesh intraoperatively. Additional larger series of studies will likely be published by our group.
